# Electrophysiological, cognitive and clinical profiles of at-risk mental state: The longitudinal Minds in Transition (MinT) study

**DOI:** 10.1371/journal.pone.0171657

**Published:** 2017-02-10

**Authors:** Rebbekah J. Atkinson, W. Ross Fulham, Patricia T. Michie, Philip B. Ward, Juanita Todd, Helen Stain, Robyn Langdon, Renate Thienel, Georgie Paulik, Gavin Cooper, MinT Consortium, Ulrich Schall

**Affiliations:** 1 Centre for Brain and Mental Health Research, University of Newcastle, Newcastle, New South Wales, Australia; 2 Hunter Medical Research Institute, Newcastle, New South Wales, Australia; 3 Schizophrenia Research Institute, Darlinghurst, New South Wales, Australia; 4 School of Psychology, University of Newcastle, Newcastle, New South Wales, Australia; 5 School of Medicine and Population Health, University of New South Wales, Sydney, New South Wales, Australia; 6 Schizophrenia Research Unit, South Western Sydney Local Health District, Sydney, New South Wales, Australia; 7 Centre for Rural and Remote Mental Health, Bloomfield Hospital, Orange, New South Wales, Australia; 8 School of Social and Health Sciences, Leeds Trinity University, Horsforth Leeds, United Kingdom; 9 ARC Centre of Excellence in Cognition and Its Disorders, Macquarie University, Sydney, New South Wales, Australia; 10 Department of Cognitive Science, Macquarie University, Sydney, New South Wales, Australia; 11 Hunter Institute for Mental Health, Newcastle, New South Wales, Australia; 12 School of Psychology, University of Western Australia, Nedlands, Western Australia, Australia; 13 School of Psychology and Exercise Science, Murdoch University, Murdoch, Western Australia, Australia; 14 Hunter New England Health, Newcastle, Australia; Chiba Daigaku, JAPAN

## Abstract

The onset of schizophrenia is typically preceded by a prodromal period lasting several years during which sub-threshold symptoms may be identified retrospectively. Clinical interviews are currently used to identify individuals who have an ultra-high risk (UHR) of developing a psychotic illness with a view to provision of interventions that prevent, delay or reduce severity of future mental health issues. The utility of bio-markers as an adjunct in the identification of UHR individuals is not yet established. Several event-related potential measures, especially mismatch-negativity (MMN), have been identified as potential biomarkers for schizophrenia. In this 12-month longitudinal study, demographic, clinical and neuropsychological data were acquired from 102 anti-psychotic naive UHR and 61 healthy controls, of whom 80 UHR and 58 controls provided valid EEG data during a passive auditory task at baseline. Despite widespread differences between UHR and controls on demographic, clinical and neuropsychological measures, MMN and P3a did not differ between these groups. Of 67 UHR at the 12-month follow-up, 7 (10%) had transitioned to a psychotic illness. The statistical power to detect differences between those who did or did not transition was limited by the lower than expected transition rate. ERPs did not predict transition, with trends in the opposite direction to that predicted. In exploratory analysis, the strongest predictors of transition were measures of verbal memory and subjective emotional disturbance.

## Introduction

The incidence rate for onset of schizophrenia is highest during late adolescence and early adulthood [1]. Long-term outcomes are heterogeneous across individuals. While some individuals experience sustained remission from symptoms, many others have lifetime reductions in quality of life associated with episodes of florid positive symptoms, as well as ongoing negative symptoms and impaired cognitive function [2]. In order to develop more effective interventions and reduce disability, it is essential to better understand the onset and course of the illness. In individuals with a confirmed diagnosis, it is usually possible to *retrospectively* identify a prodromal period lasting several years during which signs of poor mental health were evident before the first onset of frank psychosis. These indications may include (a) basic symptoms, which refer to the individual’s subjective experience of abnormal volition, emotions, thoughts, language, sensory perceptions, or motor actions; (b) attenuated psychotic symptoms, which are observable symptoms normally associated with psychosis but of reduced intensity; and (c) generalised reductions in ability to function, such as a decline in academic achievement. Internationally, a number of clinical instruments have been devised to identify individuals who are at risk of developing a psychotic illness. These instruments differ in the extent to which they monitor attenuated psychotic symptoms, basic symptoms, and functional status [3]. The Comprehensive Assessment of At Risk Mental State (CAARMS) is a structured interview designed to identify individuals considered to be at ultra-high risk (UHR) of developing psychosis [4]. Initial reports estimated that 20–35% of help-seeking clients meeting the CAARMS UHR criteria will develop schizophrenia or a schizophrenia-spectrum disorder within 12–18 months [5]. However the figure can be as high as 50% [6] depending on the population sampled, the follow-up period, and the diagnostic criteria. In principle, this high transition rate provides an opportunity to study the evolving disorder in a relatively short time frame. From a clinical perspective, improving the identification of those UHR individuals who are more likely to make a transition to schizophrenia should allow for more targeted early intervention in the prodromal phase of the illness and potentially better long-term outcomes [7].

### Mismatch negativity

All of the UHR screening instruments currently in use rely entirely on clinical interview [3]. The incorporation of objective, quantifiable, physiological measures might improve their predictive power [8, 9]. Currently, one of the most reliable markers of schizophrenia is the reduction of an electrophysiological signal recorded from the brain referred to as mismatch negativity (MMN) [10–14]. MMN is an early negative component of the event-related potential (ERP), which is elicited by any discriminable change in a repetitive background of auditory stimulation. The memory system underlying MMN enables the brain to process sounds with respect to a relevant acoustic context and to automatically identify those events that might be behaviourally relevant, prompting an attention switch for further processing [15]. This memory system incorporates a model of the acoustic context that is used to make perceptual inferences about the nature of future sound events [16].

MMN is a useful index for several reasons. MMN reduction is a robust finding in schizophrenia [13, 17]; while MMN reduction has been postulated to be a general index of cognitive decline across multiple disorders [18], it has moderate specificity for schizophrenia [10, 19] relative to disorders such as bipolar affective disorder, in which MMN is also reduced but with a smaller effect size [14]; the MMN process is largely “automatic” in nature; it does not require active attention and can be easily recorded in individuals while reading a book or watching a movie [20]. Moreover, the MMN abnormality can be linked to the underlying neurochemical (e.g. glutamate hypofunction) [21–23] and neuroanatomical abnormalities (e.g. reduction in cortical brain matter and associated cognitive deficits) [24, 25] which have been implicated in schizophrenia. Finally, reduced MMN appears to be closely associated with poor verbal memory [26] and poor overall level of functional status across psychological, social, and occupational domains [27]. These cognitive and functional deficits are persistently present in individuals with schizophrenia, whereas the clinically diagnostic positive symptoms, such as auditory hallucinations or persecutory delusions, can be episodic [28].

Although MMN can be elicited by almost any perceptible change in auditory stimulus features, MMN reduction in schizophrenia has a larger effect size for duration, rather than frequency or intensity, deviant stimuli [14]. It has been suggested that MMN in response to duration deviants may be a more reliable bio-marker for psychosis during the early phase of the illness, whereas gradually increasing deficits in MMN to frequency deviants index progression of the illness during the chronic phase [29–31]. For example, we have previously reported MMN deficits for both duration and frequency in individuals with a long duration of illness, but only found duration MMN deficits in recent-onset schizophrenia [29]. However, this proposition is challenged by recent findings of MMN amplitude reductions that are independent of deviant type in recent onset schizophrenia [32] as well as in a clinical high risk group [33].

During the course of the current longitudinal study, a growing number of reports have either compared MMN in an at-risk group to healthy controls [34–41] or additionally compared UHR individuals who transitioned to psychosis to those who did not transition [33, 42–45]. Reviews of these studies [9, 11, 31, 46, 47] and a meta-analysis [48] have suggested reduced MMN in UHR relative to healthy controls with the majority of studies reporting significant reduction in MMN to at least one type of deviant. Furthermore, in those studies that examined transition rates, reduced MMN, especially to duration deviants, provide some evidence which may help predict transition to psychosis. Unfortunately, interpretation of data comparing transition to non-transition UHR subgroups from these studies, including the current study, has been hampered by small sample sizes in the transition groups. Transition rates have been substantially lower than that predicted by the original CAARMS studies [5, 49]. Consequently, data needs to be pooled across multiple studies before definitive conclusions can be drawn. The current study is the largest to date in terms of the number of UHR participants examined.

### The P3a

Deviants in an auditory stream elicit MMN followed by a positive frontal component labelled P3a. The P3a is generally interpreted as an index of automatic reorientation of attention towards the deviant stimulus following change detection [50–52]. However, the relationship between MMN and P3a is not fully understood. For example, P3a amplitude is more sensitive to attentional load than MMN and can be abolished if the primary task is cognitively demanding [50, 53]. Originally, it was proposed that P3a is triggered when the change detection process indexed by MMN exceeds a threshold, the level of which is subject to top-down control [54]. More recently, experimental manipulations have demonstrated dissociations between MMN and P3a, which suggest P3a may be initiated independently of MMN by lower-level change detection/salience processes [55, 56], and query whether P3a actually indexes a shift of attention [56, 57]. Studies of individual differences in patient cohorts with established schizophrenia [58, 59] further support the dissociation of MMN and P3a, and argue that deficits in these reflect distinct physiological abnormalities. In line with this, pharmacological studies suggest MMN reflects glutamatergic processes [60, 61], whereas P3a amplitude is modulated by dopamine (and hence current anti-psychotic medications) [62, 63]. Consistent with the findings for MMN, P3a amplitude correlates with cognitive and psychosocial functioning [59, 64, 65], and is reduced in schizophrenia [59, 66], first episode psychosis [37, 41, 64, 67], and in at risk samples [36, 37, 39, 41]. Thus P3a might provide an additional biomarker for psychosis but targeting different disease processes, and can be readily measured concurrently with MMN. However, to date, only three studies have examined whether P3a predicts transition to psychosis in UHR individuals. Two reported reduced [36, 39] and one increased [44] P3a in those who transitioned, but in all three studies sample sizes were extremely small and the effects not statistically significant. Thus the utility of P3a as a potential biomarker for psychosis is yet to be determined.

### Cognition

A drop in global functioning is a key indicator of risk of developing psychotic illness, including schizophrenia [5]. There is some evidence that MMN amplitude reduction is associated with poor functioning, while other reports suggest that MMN amplitude reduction in patients is also related to more traditional measures of verbal memory performance and executive function subtests [26]. These more traditional cognitive measures have also been associated with functional disability. Deficits in verbal memory, verbal fluency, and executive functioning/working memory have been reported in UHR samples [68]. However, many past studies have used composite test batteries and do not report data on specific deficits on individual subtests. Where subtests have been examined, these have revealed preliminary evidence that Logical Memory from the WMS-R and spatial working memory may be differentially impaired in those who later develop psychosis [68]. Previous reports also suggested that olfactory identification deficits appear to be predictive of transition to psychosis and even specific to those UHR individuals who developed schizophrenia compared to those who developed other forms of psychoses outside of the schizophrenia spectrum [69]. However, more recent reports suggest that poor olfactory identification is associated with poor functional outcome, regardless of transition status [70].

While real-world functional disability in schizophrenia is associated with various cognitive deficits, to a degree, there is growing evidence that specific “socio-cognitive” impairments are more closely associated with the poor daily social interactions that characterise schizophrenia. “Socio-cognitive” refers to the processes that high-order primates, including human beings, have evolved to sustain complex social interactions. One of the most important of these is Theory of Mind (ToM); that is the capacity to use contextual cues to reason about, predict, and explain behaviour in terms of psychological causation (e.g., to reason that “Fred desires x and intends y since he believes z about this situation”). ToM is severely impaired in schizophrenia [71, 72]. ToM also better predicts real world functioning in people with schizophrenia than do more basic cognitive abilities like attention [73]. The current study assessed ToM using three tasks, two of which make minimal verbal demands: (1) the non-verbal False-belief Picture-sequencing Task [74], (2) the Reading the Minds in the Eyes Task (with minimal verbal demands) [75], and (3) the language-based Hinting Task [76].

### Aims of the study

In the current longitudinal study, a cohort of help-seeking individuals identified to be at risk of developing a psychotic illness was examined with the intention of addressing several specific questions. Firstly, do those at risk of developing psychosis differ from a healthy cohort, particularly in their MMN response? Does MMN amplitude differ between those UHR participants who transition to psychosis and those who do not? Is transition to psychosis associated with further reductions in MMN amplitude after the baseline measurement? Is the reduction in MMN associated with changes in functional status and cognitive impairment especially in verbal memory? Finally, is MMN in response to duration-deviants a more robust predictor of transition rates than MMN to frequency or intensity deviants? We also take the opportunity to ask similar questions of the P3a which immediately follows the MMN during a passive auditory task, and examine its relationship with MMN. Finally, we examined a battery of clinical and cognitive measures within the UHR sample and their relationship with MMN.

## Method

### Ethics

Ethics approval was provided by the Hunter New England Human Research Ethics Committee (Approval number: 08/12/17/5.17). All participants provided written informed consent. For participants under 18 years of age, a parent or guardian also provided written informed consent. Participants received monetary compensation to cover travelling expenses. At the participant’s discretion, referring clinicians received a report summarising clinically relevant test results.

### Participants

Data presented are from the Minds in Transition (MinT) project (2009–2014), which is a longitudinal study of transition to schizophrenia in UHR participants. The research was conducted in collaboration with early psychosis services in metropolitan, regional, and rural centres in NSW Australia (The Schizophrenia Research Unit, Liverpool Hospital, South Western Sydney and the Early Psychosis Program, Bondi Junction Community Mental Health Service, Eastern Sydney; The Psychological Assistance Service, Hunter New England Health and the Centre for Translational Neuroscience and Mental Health Research, The University of Newcastle, Newcastle; and the Centre for Rural and Remote Mental Health, Bloomfield Hospital, Orange; respectively). Participant referrals were variously obtained through the national headspace initiative, mental health workers, general practitioners, school counsellors and self-referrals.

UHR participants were aged 13–25 years. Inclusion criteria required a loss of functioning, defined as a drop of 30% in Global Assessment of Functioning (GAF) in the past 12 months, together with at least one of the following: (a) schizotypal personality traits or a first degree relative with schizophrenia; (b) attenuated psychotic symptoms; or (c) brief limited intermittent psychotic symptoms (BLIPS). Criteria b and c are assessed on the CAARMS [4], an interview schedule designed to assess a broad range of sub-threshold symptoms commonly reported in prodromal psychosis. Exclusion criteria for the study included pre-existing psychosis with symptoms exceeding the CAARMS psychosis threshold, antipsychotic pharmacotherapy, diagnosis of drug abuse or dependence as assessed by either the Structured Clinical Interview for DSM-IV Axis I Disorders–Clinical Version (SCID-CV) or the Kiddie Schedule for Affective Disorders and Schizophrenia for School-aged Children—Present and Lifetime version (K-SADS-PL), head injury with loss of consciousness for more than 15 mins, organic brain impairment, estimated pre-morbid IQ of less than 70, impaired hearing (>20dB SPL), or history of nasal trauma.

Cohorts of healthy comparison participants were recruited from the general community at each of the three research sites (i.e. Sydney, Orange and Newcastle) using a variety of procedures including research volunteer registers; newspaper, website and notice board adverts; and word of mouth advertising within hospital and educational institutions. We did not actively recruit friends or relatives of UHR participants. Recruitment procedures were aimed to provide Control and UHR groups with similar age and gender profiles. Additional exclusion criteria for Controls included having a first-degree family member with schizophrenia, or a history of treatment for depression or anxiety.

After initial screening, 102 UHR and 61 healthy comparison participants were recruited. Some participants did not complete a baseline EEG recording (18 UHR) or their EEG recording was excluded due to data quality issues (4 UHR, 3 Control). The remaining participants consisted of 80 UHR (42 females, mean age 18.6 yrs) and 58 controls (28 females, mean age 19.1 yrs) ranging in age from 12 to 26 years.

### General procedure

At baseline, over the course of 2–3 days, all participants undertook a battery of clinical and neuropsychological tests, an EEG recording whilst performing a passive and an active auditory task, and a structural MRI session. Controls did not participate in any follow-up testing. UHR participants were contacted every three months for the first year to assess their clinical status. At the 12-month follow-up, the clinical tests, a subset of the neuropsychological tests, the EEG recording, and the MRI session were repeated. A sub-group of UHR participants, excluding those at the rural test centre, were monitored at six-monthly intervals for a further two years. At the conclusion of the longitudinal study, participants were classified as either healthy comparison participants (Control); UHR participants who were lost to follow-up prior to the 12-month follow-up session (LTFU); UHR who had not transitioned to psychosis (UHR-NT); or UHR who had transitioned to psychosis (UHR-T). Transition to psychosis was confirmed by a DSM-IV diagnosis on either the SCID-CV or K-SADS-PL.

### Clinical assessment

At baseline, clinical assessment began with a patient history, including family mental health, past treatments, and prescription medications. Current diagnosis was assessed using the SCID-CV [77] for participants 18 years or older, or the K-SADS-PL [78] for participants under 18 years. Current mental health status and symptoms were assessed using the Comprehensive Assessment of At Risk Mental States–Monthly Version 2006 (CAARMS) [4], and the Brief Psychiatric Rating Scale (BPRS) [79]. The impact of mental health problems was assessed using the Global Assessment of Functioning (GAF) from the DSM-IV, the Social and Occupational Function Assessment Scale (SOFAS) [80], and the Global Functioning: Social (GF: Social) and Global Functioning: Role (GF: Role) scales [81]. Substance use was assessed with the Alcohol Use Disorders Identification Test (AUDIT) [82], the Cannabis Use Disorders Identification Test (CUDIT) [83], and the Opiate Treatment Index: drug use all types (OTI) [84]. Other clinically relevant assessments included the Schizotypal Personality Questionnaire (SPQ) [85], the Rosenberg Self Esteem Scale (RSES) [86], the Beck Depression Inventory II (BDI-II) [87], the Beck Anxiety Inventory (BAI) [88], the Eysenck Personality Questionnaire–Revised (EPQ-R) [89], and the Pittsburgh Sleep Quality Index (PSQI) [90].

Follow-up assessments conducted every 3 months for the first year, and 6 monthly thereafter, included current medication and treatment, symptom ratings using the BPRS, global functioning (GAF, SOFAS, GF: Social, GF: Role), alcohol and drug use, AUDIT and CUDIT. The full battery of clinical assessments was repeated at the 12-month follow-up.

### Neuropsychological assessment

At baseline, Premorbid IQ [91] was estimated with the two subtest (Vocabulary and Matrix Reasoning) version of the Wechsler Abbreviated Scale of Intelligence (WASI) [92]. Working memory was assessed with the Digit Span and Letter Number Sequencing sub-tests from the Wechsler Memory Scale (WMS-III) [93] for participants 17 years or older, and from the Wechsler Intelligence Scale for Children (WISC-IV) [94] for participants under 17 years. Episodic verbal learning and memory was assessed with the California Verbal Learning Task (CVLT-II) [95] for participants 16 years or older, or the California Verbal Learning Test for Children (CVLT-C) [96] for participants under 16 years. Short-term visual memory was assessed with the Visual Patterns Test (VPT) [97]. Executive function was assessed with the Trail Making, Verbal Fluency, Colour Word Interference, and Tower Tests of the Delis-Kaplan Executive Function System (D-KEFS) [98]. Social cognition was assessed with the False-Belief Picture-Sequencing Task (FBPST) [75], Reading the Mind in the Eyes Task, revised version (RMET) [76], and the Hinting Task [99]. Integrity of olfactory perception was assessed with the University of Pennsylvania Smell Identification Task (UPSIT) [100].

### Passive auditory task

For the passive auditory task, participants watched a video with muted audio while binaural auditory stimuli were presented using calibrated headphones. There were four auditory stimuli including a standard tone (50 ms, 80 dB SPL, 1 kHz sine wave, 10 ms rise and fall times), and three deviant tones that differed from the standard in one physical feature: duration (100 ms), frequency (1.2 kHz) or intensity (90 dB). Stimuli were presented with 500 ms stimulus onset asynchrony in 3 blocks of trials each lasting approximately 9 minutes, with rest breaks between blocks. Each block began with 3 sub-blocks of 80 trials of one of the three deviant types, presented as a control for physical features of the deviant stimuli. This was followed by an oddball sequence of 860 tones consisting of the standard (82%) and three deviants (6% each) in a pseudo-random order, such that each deviant was preceded by a minimum of two standards.

The passive auditory task was always followed by an active auditory task, the results of which will be presented elsewhere.

### EEG acquisition

EEG data were acquired from nine scalp sites (F3, Fz, F4, C3, Cz, C4, P3, Pz, P4) and both mastoids, referenced to the tip of the Nose, using an electrode cap (Quick Cap, Neuroscan). Vertical EOG was recorded from electrodes above and below the left eye. Horizontal EOG was recorded from electrodes adjacent to the outer canthi of both eyes. A ground electrode was located at AFz. Data were sampled at 500 Hz.

EEG data were recorded on one of two EEG Amplifier systems, either a Neuroscan QuickAmps or Neuroscan Synamps II. These EEG systems have different digital filtering characteristics. Consequently, after acquisition, EEG data from both systems were additionally filtered to compensate for these differences, such that the final EEG from both systems was identically stimulus-locked, calibrated, and band pass filtered (0.5 to 30 Hz; 50 Hz notch).

### ERP processing

ERP extraction was performed using Neuroscan Edit v4.5 software. Visual inspection was performed to exclude gross artifact and identify bad channels. Bad channels were replaced by interpolation of adjacent sites. Blink related artifact was reduced using a linear regression procedure with regression weights derived from an averaged blink [101].

For the passive auditory task, the EEG was low-pass filtered at 30 Hz and epoched from 100 ms pre-stimulus to 450 ms post-stimulus. Within the oddball sequence, the first 10 stimuli and the first standard following each deviant were excluded. Trials with EEG artifact exceeding ±150 μV were excluded. Trials were baseline corrected relative to the 100 ms pre-stimulus interval; re-referenced to the average of both mastoids; and averaged by stimulus type to produce ERPs to the Common Standard (STD), Duration Deviant (DEV_Dur_), Frequency Deviant (DEV_Frq_) and Intensity Deviant (DEV_Int_) stimuli. For each of the three deviant types, MMN difference waves (MMN_Dur_, MMN_Frq_, MMN_Int_) were computed by subtracting the STD from the corresponding Deviant ERP. The MMN difference waves were additionally low-pass filtered at 20 Hz.

### ERP measures

Preliminary analysis indicated significant age related shifts in ERP peak latencies. Hence we estimated peak amplitude and latency measures rather than computing mean amplitudes across fixed time intervals. Peak latencies were estimated at Fz for all ERP components using the search intervals listed in Table 1. Search intervals were centred on the peak response within the grand average ERP across all participants and were broad enough to capture peaks for all individuals as confirmed by visual inspection. For each participant, peak latency was estimated using the 50% fractional area latency (FA-latency) method. FA-latency is more robust than simple peak measures [102]. Peak amplitude measures were then defined at that latency, as the mean across a 50 ms window centred on the peak.

**Table 1:** Peak Latency of ERP Components in the Passive Auditory Task.

Component	Condition	Peak Latency	Search Window
**MMN**	Duration	186	116–256
	Frequency	168	98–238
	Intensity	158	88–228
**P3a**	Duration	282	192–372
	Frequency	272	182–362
	Intensity	268	178–358

Peak latency for each ERP component of interest in the grand average ERP (across all participants at baseline) and the intervals searched when measuring each participant’s peak amplitude and latency for statistical analyses.

### Statistical procedures

Each ERP measure was analysed independently. To assess differences between UHR and controls at baseline, ANCOVA was performed with factors group (Control, UHR) and deviant-type (Duration, Frequency, Intensity) co-varied for age. For comparability with previous studies, tables present results of separate ANCOVAs for each deviant type including means, standard deviations and effect sizes reported as Cohen’s *d*. To assess UHR sub-groups at baseline, the ANCOVA was repeated, but with four groups (Control, LTFU, UHR-T, UHR-NT), co-varied for age, followed by planned comparisons between UHR-T and each of the other three groups. Only effects involving group are reported. To look at differential changes in ERP components over time, a longitudinal ANCOVA was performed across the two groups who provided data at both baseline and the 12-month follow-up (UHR-T, UHR-NT), with Session (Baseline, Follow-up) as an additional repeated measures factor, co-varied for age. Only effects involving group are reported. Greenhouse-Geisser correction was performed for repeated measures factors. An alpha of 0.05 (two-sided) was accepted as statistically significant for hypothesis testing. Where relevant, the term MMN_mean_ refers to the simple average of MMN_Dur_, MMN_Frq_ and MMN_Int_. Similarly for P3a_mean_.

Items in the battery of clinical and neuropsychological measures were contrasted across groups using ANCOVA co-varied for age. The relationships between MMN and P3a with clinical measures were examined using partial correlations controlling for age. To control for multiple comparisons, all results with uncorrected *p* < .005 were accepted as significant, we then applied a false discovery rate of α = .05 to the remaining variables.

## Results

### Transition to psychosis

At the conclusion of the longitudinal study, participants were classified as either healthy comparison participants (Control, *n* = 61); UHR participants who were lost to follow-up prior to the 12-month follow-up session (LTFU, *n* = 35); UHR who had not transitioned to psychosis (UHR-NT, *n* = 60); or UHR who had transitioned to psychosis (UHR-T, *n* = 7). Transition to psychosis was confirmed by a DSM-IV diagnosis on either the SCID or KSADS. DSM-IV diagnoses included 1 x Paranoid Schizophrenia, 2 x Psychotic Disorder (NOS), 1 x Schizoaffective Disorder, 2 x Bipolar I Disorder with Psychotic Features, and 1 x Major Depressive Disorder with Psychotic Features.

### Demographic, clinical, and neuropsychological measures at baseline

A summary of key demographic measures contrasting controls and UHR are presented in Table 2. The full set of demographic, clinical and neuropsychological measures and statistical comparisons are presented in S1 Table. As expected, controls and UHR differed substantially on a wide range of measures after controlling for effects of age. Controls and UHR did not differ on age, gender or handedness. However, UHR were significantly (FDR < .05) less likely to be engaged in employment, secondary or tertiary education; had two years less education than controls; had a greater chance of having a relative with mental health issues; had poorer general functioning; substantially higher rates of previous mental health issues, especially depression (72%), anxiety (56%), drug/alcohol related problems (35%), self-harm (37%) and attempted suicide (27%); were substantially more likely to be using prescription medications (52%), especially antidepressants (33%); were more likely to have a history of tobacco or illegal drug usage; had first used cannabis two years earlier; and reported greater impacts from both alcohol and cannabis use. 61% of UHR self-reported having tried one or more illicit drugs and 45% reported a history of regular drug use; compared to 31% and 8% of controls respectively. 25% of UHR and 7% of controls reported consuming an illicit drug in the week preceding testing. Cannabis was the most frequently consumed drug, accounting for approximately half of all reports, though poly drug use was also high. UHR were significantly different from controls, in the expected direction, on clinical measures using the SPQ, RSES, BDI-II, BAI, EPQ-R, and PSQI.

**Table 2:** Key demographic measures contrasting UHR and controls at baseline.

Measure	Sub-Measure	Control	UHR	Statistic	Significance	Cohen’s *d*
*n*		61	102			
Age		19.1 (3.19)	18.6 (2.71)	*t*(161) = 1.04	n.s.	0.168
Gender	Male	32	47	χ ^2^(1) = .62	n.s.	0.124
	Female	29	55			
Handedness	Right	48	83	χ ^2^(2) = .30	n.s.	
	Left	5	7			
	Ambidextrous	6	8			
Employment	Employed/Student	57	67	χ ^2^(1) = 21.9	*p* < .001 **	0.799
	Unemployed	1	34			
Years of Education		11.9 (2.62)	10.0 (2.54)	*F*(1,160) = 26.0	*p* < .001 **	0.807
Medication	Any Medication †	4	46	χ ^2^(1) = 26.6	*p* < .001 **	0.922
	Nil	52	50			
Previously Treated Mental Health Problems	Any	2	90	χ ^2^(1) = 114.2	*p* < .001 **	3.193
	Nil	57	10			
Family History (First Degree)	Schizophrenia ††		24	χ ^2^(1) = 16.7	*p* < .001 **	0.685
	Nil	59	76			
Cannabis Use	Have Used	19	58	χ ^2^(1) = 12.8	*p* < .001 **	0.596
	Have Never Used	42	38			
	Age First Used	16.5 (1.81)	14.8 (2.27)	*F*(1,73) = 7.60	*p* = .007 **	0.645
	Age Regular Usage	16.6 (3.21)	15.2 (1.72)	*F*(1,39) = 1.78	*p* = .193	0.425
	Duration Use (users)	2.58 (2.59)	3.50 (2.62)	*F*(1,74) = 6.61	*p* = .012 *	0.598
	Duration Use (all)	0.80 (1.86)	2.12 (2.66)	*F*(1,154) = 17.0	*p* < .001 **	0.664
WASI 2 Subscale IQ		118 (12.0)	104 (16.5)	*F*(1,153) = 34.2	*p* < .001 **	0.946
Global Assessment of Functioning		85.7 (6.12)	55.5 (12.2)	*F*(1,145) = 260	*p* < .001 **	2.678

Means (Standard Deviation) and statistical significance of the difference between Controls and UHR after co-varying for age. For clarity, p values >.1 listed as n.s. Effect size reported as Cohen’s d.

† Antipsychotic medication was one of the exclusion criteria. 4 Controls and 13 UHR were taking non-psychotropic/non-specified medications.

†† 1^st^ degree family history of schizophrenia was an exclusion criteria for controls.

* *p* < .05 uncorrected.

** *p* < .01 uncorrected.

Results were mixed on tests of neuropsychological performance (S1 Table). Relative to controls, UHR had lower IQ (WASI), working memory (letter-number sequencing, digit span), short term visual memory (VPT), and verbal memory (CVLT). On tests of executive function (D-KEFS), UHR were impaired on the verbal fluency, colour-word interference, and trail making tasks but not on the tower-test. Results for tests of social cognition were mixed, with impaired performance on the Hinting Task, but not on the False-Belief Picture Sequencing task or Reading the Mind in the Eyes Task. Finally, UHR were not impaired on the University of Pennsylvania Smell Identification Task.

There were relatively few differences between UHR-T (*n* = 7) and UHR-NT (*n* = 60) sub-groups. S2 Table lists statistical comparisons on all measures, including mean (*SD*) and effect size. There were no effects of age or gender. After adjusting for age, UHR-T differed from UHR-NT in the expected direction, on the CAARMS Subjective Emotional Disturbance sub-scale (Intensity*Frequency; *M*_UHR-T_ = 17.8, *SD* = 7.22, *M*_UHR-NT_ = 7.53, *SD* = 6.85), *F*(1,63) = 11.4, *p* = .001, *d* = .85; and the CVLT-II Item Recognition sub-scale (*M*_UHR-T_ = -1.29, *SD* = 1.82, *M*_UHR-NT_ = -.242, *SD* = .696), *F*(1,62) = 8.29, *p* = .005, *d* = .73. Additionally, there were trends (uncorrected *p* < .05) in the same direction for the CVLT-II Delayed Recall and the Global Functioning Role scales.

### Event-related potentials: UHR vs healthy controls

**Fig 1:**
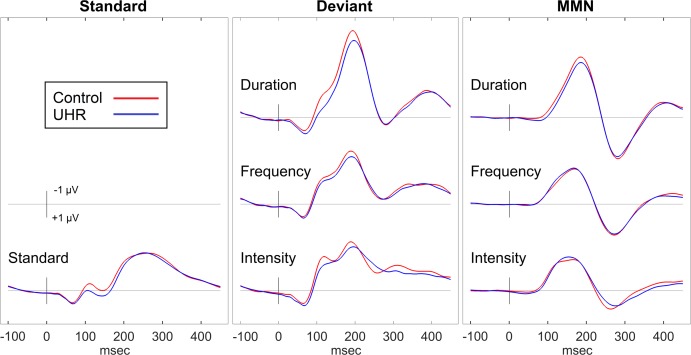
Comparison of Control and UHR Groups. ERP waveforms for the passive auditory task at baseline contrasting the response from the Control and UHR groups.

At baseline, EEG data was obtained from 58 Control and 80 UHR. There were no significant differences between groups on age or gender. Fig 1 contrasts the ERP waveforms for the control and UHR groups.

An omnibus ANCOVA across the three deviant types (Duration, Frequency, Intensity), co-varied for age, revealed no significant differences between UHR and Control groups on any of the ERP amplitude [MMN: *F*(1,135) = .02; P3a: *F*(1,135) = .02] or latency measures [MMN: *F*(1,135) = .09; P3a: *F*(1,135) = 1.09]. There were no interaction effects between group and deviant type. For both ERP components, increasing age was significantly associated with earlier peak latency [MMN: *F*(1,135) = 14.6, *p* < .001; P3a: *F*(1,135) = 5.43, *p* = .021] and larger peak amplitude [MMN: *F*(1,135) = 6.37, *p* = .013; P3a: *F*(1,135) = 5.72, *p* = .018]. Means, standard deviations and effect sizes for the corresponding ERP measures are presented in Table 3.

**Table 3:** Comparison of UHR and Control ERPs for the passive auditory task.

		Control	UHR	Statistic	*p*	Cohen’s *d*
*n*		58	80			
Age		19.1 (3.18)	18.6 (2.68)	*t*(136) = .96	.34	
Male/Female		30/28	38/42	χ^2^(1) = .24	.62	
MMN_Amplitude_						
	Duration	-4.80 (2.12)	-4.49 (2.28)	*F*(1,135) = .32	.57	.098
	Frequency	-3.00 (1.66)	-3.13 (1.58)	*F*(1,135) = .33	.57	.099
	Intensity	-3.01 (1.52)	-3.14 (1.96)	*F*(1,135) = .30	.58	.095
MMN_Latency_						
	Duration	183.6 (13.3)	185.5 (16.9)	*F*(1,135) = .32	.58	.097
	Frequency	164.3 (19.8)	163.3 (24.0)	*F*(1,135) = .29	.59	.093
	Intensity	160.1 (27.9)	159.3 (26.4)	*F*(1,135) = .12	.74	.059
P3a_Amplitude_						
	Duration	3.27 (2.17)	3.32 (1.93)	*F*(1,135) = .15	.70	.066
	Frequency	2.59 (1.68)	2.54 (2.01)	*F*(1,135) < .01	.99	.003
	Intensity	1.82 (1.78)	1.79 (1.75)	*F*(1,135) < .01	.96	.010
P3a_Latency_						
	Duration	287.9 (18.3)	286.8 (21.2)	*F*(1,135) = .18	.67	.074
	Frequency	274.8 (21.8)	277.3 (21.2)	*F*(1,135) = .19	.67	.075
	Intensity	269.4 (27.5)	279.4 (31.5)	*F*(1,135) = 3.42	.07	.318

Means, standard deviations and effect sizes for the comparison of ERP measures in UHR and controls at baseline. Mean (*SD*) are raw scores in μV or ms. *F*, *p* and *d* statistics are from an ANCOVA co-varied for age (performed separately for each deviant type for MMN and P3a measures). There are no statistically significant differences between UHR and control groups in this table.

### Event-related potentials: UHR-T vs control, UHR-NT and LTFU

In total, there were 61 Controls, 7 UHR-T, 60 UHR-NT, and 35 LTFU. Of these, baseline EEG data was available for the passive auditory task from 58 Control, 6 UHR-T, 55 UHR-NT, and 19 LTFU. There were no age or gender differences between groups. Given the small sample size in the UHR-T group, statistical comparisons need to be interpreted with caution due to low statistical power.

**Fig 2:**
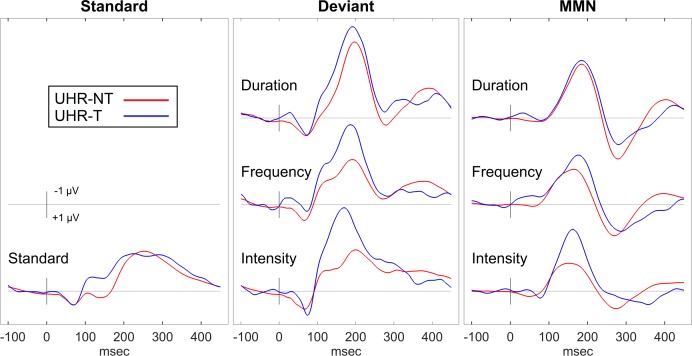
Comparison of UHR-T and UHR-NT Groups. ERP waveforms for the passive auditory task at baseline contrasting the response from the UHR-T and UHR-NT groups.

Fig 2 presents ERP waveforms contrasting the UHR-T and UHR-NT groups at baseline. Contrary to expectations, UHR-T tended to have larger MMN responses than UHR-NT. Means, standard deviations and effect sizes for ERP measures in Control, LTFU, UHR-NT and UHR-T groups at baseline are provided in Table 4.

**Table 4:** Comparison of UHR sub-groups’ ERPs for the Passive Auditory Task.

	Control	LTFU	UHR-NT	UHR-T	UHR-T—Control			UHR-T—UHR-NT		
					Stats	*p*	*d*	Stats	*p*	*d*
**N**	58	19	55	6						
**Age**	19.1 (3.19)	18.8 (2.97)	18.4 (2.62)	19.8 (1.96)	t_134_ = .39	.70	.166	t_134_ = .91	.37	.391
**Male/Female**	30/28	10/9	25/30	3/3	χ^2^(1) = .006	.94		χ^2^(1) = .05	.83	
**MMN Amplitude**										
Duration	-4.80 (2.12)	-4.54 (2.32)	-4.43 (2.28)	-4.95 (2.41)	t_133_ = .07	.95	.029	t_133_ = .33	.74	.142
Frequency	-3.00 (1.66)	-3.09 (1.91)	-3.07 (1.38)	-3.77 (2.21)	t_133_ = 1.07	.29	.460	t_133_ = .91	.37	.390
Intensity	-3.01 (1.52)	-3.67 (1.76)	-2.78 (1.94)	-4.81 (1.69)	t_133_ = .2.37	.019 *	1.014	t_133_ = 2.61	.010 *	1.121
**MMN Latency**										
Duration	183.6 (13.3)	189.1 (15.8)	184.8 (17.3)	181.1 (17.3)	t_133_ = .32	.75	.136	t_133_ = .41	.69	.174
Frequency	164.3 (19.8)	166.8 (23.1)	160.9 (24.6)	173.8 (19.2)	t_133_ = 1.15	.25	.493	t_133_ = 1.67	.10	.716
Intensity	160.1 (27.9)	155.4 (21.4)	160.4 (29.0)	162.2 (16.5)	t_133_ = .25	.80	.107	t_133_ = .32	.75	.138
**P3a Amplitude**										
Duration	3.27 (2.17)	3.26 (1.66)	3.43 (1.92)	2.46 (2.88)	t_133_ = 1.06	.29	.455	t_133_ = 1.39	.17	.595
Frequency	2.59 (1.69)	2.40 (1.36)	2.60 (2.06)	2.43 (3.38)	t_133_ = .26	.79	.113	t_133_ = .35	.73	.152
Intensity	1.82 (1.78)	1.42 (1.41)	1.90 (1.87)	1.97 (1.66)	t_133_ = .18	.86	.077	t_133_ = .05	.96	.022
**P3a Latency**										
Duration	287.9 (18.3)	290.8 (21.0)	285.2 (19.6)	289.2 (35.6)	t_133_ = .21	.84	.088	t_133_ = .59	.56	.252
Frequency	274.8 (21.8)	280.8 (21.8)	274.2 (19.8)	294.4 (25.0)	t_133_ = 2.36	.020 *	1.01	t_133_ = 2.57	.011 *	1.11
Intensity	269.4 (27.5)	292.8 (34.0)	273.8 (27.9)	288.8 (45.2)	t_133_ = 1.58	.12	.677	t_133_ = 1.28	.20	.551

Means, standard deviations and effect sizes for the comparison of ERP measures in UHR-T to UHR-NT, control and LTFU groups at baseline. Means (*SD*) are raw scores in μV or ms. *t*, *p* and Cohen’s *d* statistics are from planned contrasts following an ANCOVA co-varied for age performed separately for each deviant type. Data in this table are presented only for comparison with prior studies. Statistical analysis within the text is based upon an omnibus ANCOVA which includes deviant type as a factor.

* *p* < .05.

For MMN peak amplitude, an omnibus ANCOVA across the three deviant types (Duration, Frequency, Intensity) co-varied for age, revealed no overall significant effect of group, *F*(3,133) = .86, n.s. The interaction between deviant type and group was not significant, *F*(6,266) = 1.44, n.s. Contrary to predictions, the UHR-T group had the largest MMN, but there were no significant differences in planned comparisons between UHR-T and the Control, *t*(133) = 1.35, *p* = .18, *d* = .58; LTFU, *t*(133) = .96, *p* = .34, *d* = .45; or UHR-NT, *t*(133) = 1.51, *p* = .13, *d* = .65, groups. There were no group effects for MMN latency.

For P3a peak amplitude, there was no significant effect of group, F(3,133) = .37, n.s., or interaction between group and deviant type. F(6,266) = .47, n.s. For P3a peak latency, there was an overall effect of group, *F*(3,133) = 3.05, *p* = .031. The UHR-T and LTFU groups had slower P3a than the Control and UHR-NT groups, but trends in the planned comparisons between UHR-T (M = 290.8, *SD* = 30.0 ms) and UHR-NT (M = 277.7, *SD* = 18.1 ms, *t*(133) = 1.94, *p* = .054, *d* = .835); Control (M = 277.4, *SD* = 16.3 ms, *t*(133) = 1.88, *p* = .062, *d* = .807); or LTFU (M = 288.1, *SD* = 16.8 ms, *t*(133) = .45, *p* = .65, *d* = .212) groups failed to reach significance.

To permit direct comparison of these ERP data with previous reports, Table 4 presents the means and standard deviations of each ERP measure for controls, UHR-T, UHR-NT and LTFU groups, with contrasts and effect sizes computed independently for each deviant type. These contrasts suggest that UHR-T had larger MMN_Int_ than both UHR-NT and controls; and UHR-T had slower P3a_Frq_ than both UHR-NT and Controls. However, these effects were not significant when corrected for multiple comparisons.

### Event-related potentials: Longitudinal analysis of UHR-T vs UHR-NT

For the longitudinal analysis, EEG data was available at both baseline and 12-month follow-up from 5 UHR-T and 44 UHR-NT. Again, the small sample size of the UHR-T group means that statistical comparisons are under-powered and results should be interpreted with due caution.

ANCOVA with repeated measures for session (baseline, follow-up) and deviant type (Duration, Frequency, Intensity) co-varied for age, indicated that MMN peak amplitude was unexpectedly larger in the UHR-T (*M* = -5.18, *n* = 5, *SD* = 2.16) than UHR-NT group (*M* = -3.36, *n* = 44, *SD* = 1.44), *F*(1,46) = 6.40, *p* = .015. The predicted interaction between group and session was not significant, *F*(1,46) = 2.25, *p* = .14. There were no group effects for MMN peak latency, P3a peak amplitude or P3a peak latency.

12-month test-retest correlations for each ERP measure in the UHR group are provided in Table 5. All peak amplitude and latency correlations were significant with medium to large effect sizes.

**Table 5:** Test-Retest Correlations for the Passive Auditory Task.

		Correlation	
	Component	Amplitude	Latency
Duration			
	MMN	.67 **	.53 **
	P3a	.49 **	.62 **
Frequency			
	MMN	.56 **	.41 *
	P3a	.62 **	.76 **
Intensity			
	MMN	.73 **	.54 **
	P3a	.39 *	.78 **

Pearson test-retest correlations for ERP measures from baseline and follow-up sessions for UHR participants. *n* = 49 for the Passive Auditory Task.

* *p* < .01.

** *p* < .001.

### Relationship between MMN, P3a and clinical measures in UHR at baseline

Given the large number of variables examined, there were no relationships between MMN or P3a and clinical measures that survived correction for multiple comparisons. Within the UHR group, as a trend, MMN_mean_ was smaller in individuals who meet the CAARMS attenuated psychosis UHR criterion (*M* = -3.39, *S*.*D*. = 1.56, *n* = 62) than those who did not (*M* = -4.25, *S*.*D*. = 1.62, *n* = 18), F(1,77) = 4.46, *p* = .038, *d* = .482. There was no relationship between MMN_mean_ amplitude and CAARMS Vulnerability or BLIP inclusion criteria, family history of psychosis (Control, UHR with, or UHR without 1^st^ degree relative with psychosis), or primary SCID or KSAD diagnosis (Control, Mood Disorder, Anxiety Disorder, No Diagnosis). Within the UHR group, we examined partial correlations, adjusting for age, between MMN_mean_ and clinical measures (S3 Table). There were trends for smaller MMN_mean_ to correlate with increased impact from cannabis use (CUDIT), *ρ* = .249, *p* = .028; and larger MMN_mean_ with higher depression scores (BDI-II), *ρ* = -.240, *p* = .035. There were no other correlations between MMN_mean_ and functional status as assessed by the GAF, SOFAS, GF-Social, or GF-Role; clinical status as assessed by the CAARMS, BPRS, SPQ, RSES, BAI, EPQ-R or PSQI; current medication; or illicit drug use including cannabis.

There was no relationship between P3a_mean_ amplitude and CAARMS inclusion criteria, family history of psychosis, primary SCID or KSAD diagnosis, or the clinical measures presented in S4 Table. The exception being a trend for smaller P3a_mean_ to be associated with larger CAARMS total Positive Symptoms, *ρ* = -.275, *p* = .014. Further exploratory analysis revealed a similar relationship between P3a_mean_ and the BPRS Hallucination subscale, *ρ* = -.325, *p* = .005.

### Relationship between MMN and P3a

The partial correlation between MMN_mean_ and P3a_mean_, adjusted for age, was significant in the UHR group, *ρ* = -.423, *n* = 79, *p* < .001; but not in Controls, *ρ* = -.060, *n* = 57, n.s. After applying Fisher’s *r* to *z* transform [103], the two group correlations were significantly different, *z* = 2.2, *p* = .028. Examining MMN and P3a separately for each deviant type, again there were no significant correlations within the Control group. In UHR, the correlation was significant for duration deviants, *ρ* = -.362, *n* = 79, *p* = .001; a trend for frequency deviants, *ρ* = -.209, *n* = 79, *p* = .064; and not significant for intensity deviants, *ρ* = -.068, *n* = 79, n.s. Applying a test for dependent correlations [103], the correlation for duration deviants was significantly larger than for intensity deviants, *z* = 2.18, *p* = .029. The correlation for frequency deviants was intermediate between but not significantly different from that for duration and intensity.

### Follow-up: Dropout rates

There were 102 UHR at baseline. Baseline testing was spread across several days with some UHR withdrawing after the neuropsychological tests, but before completing the first EEG session (*n* = 10). Additional UHR withdrew or became uncontactable before the 12-month follow-up (*n* = 25). Additionally, some EEG recordings were not conducted (Baseline: *n* = 7; Follow-up: *n* = 7) or were rejected on technical or data quality issues (Baseline: *n* = 6; Follow-up: *n* = 8). In all, 67 UHR (66%) participated at the 12-month follow-up, with 49 providing usable EEG data at both baseline and follow-up sessions.

S5 Table provides detailed statistical comparisons of UHR who were lost to follow-up prior to the 12-month follow-up. LTFU did not differ from participating UHR on age or gender, but at baseline had lower functional status [GF-Role: *F*(1,94) = 11.1, *p* = .001; GF-Social: *F*(1,94) = 5.87, *p* = .017; SOFAS: *F*(1,96) = 4.80, *p* = .031]; were more likely to have tried various drugs [cannabis: χ ^2^(1) = 8.71, *p* = .003; tobacco: χ ^2^(1) = 7.05, *p* = .008; heroin: χ ^2^(1) = 4.93, *p* = .026; amphetamines: χ ^2^(1) = 4.18, *p* = .041]; and had some cognitive impairments [D-KEFS Verbal Fluency: *F*(1,93) = 8.71, *p* = .004; D-KEFS Trail Making: *F*(1,93) = 4.15, *p* = .045; CVLT Immediate Recall: F(1,88) = 4.02, p = .048]. There were no differences on clinical measures including CAARMS, BPRS, or primary diagnosis. There were no differences in ERP amplitude measures [MMN: *F*(1,77) = .25, n.s.; P3a: *F*(1,77) = .46, n.s.].

## Discussion

Although control and UHR groups differed on a wide range of clinical and neuropsychological measures, there was no group difference in MMN or P3a amplitude. Previous studies that have compared healthy controls to UHR, without differentiating those who do or do not transition, had effect sizes for MMN reduction ranging from -.21 [41] to 1.19 [37]. Our effect sizes are certainly at the lower end of this range, but should not be considered outliers. Of the previous studies, eight reported reduced MMN in UHR relative to healthy controls [33–36, 39, 43, 45, 104], four reported no significant difference [40–42, 44], and one found a difference for duration but not frequency MMN [37]. The only modest replicability in previous studies of reduced MMN in UHR should not be too surprising. Of the UHR group a recent meta-analysis [105] estimates that less than 30% will transition to psychosis within 1–2 years, and of these, only three quarters will actually develop a schizophrenic psychosis (schizophrenia, schizophreniform disorder, or schizoaffective disorder); with another 11% developing an affective psychosis (depression with psychotic features, bipolar disorder with psychotic features); and the remainder developing other psychoses (psychosis NOS, brief psychotic episode, delusional disorder). MMN reduction shows some specificity for the cognitive deficits seen in schizophrenia relative to both bipolar affective disorder and major mood disorders irrespective of presence of psychotic symptoms [106], so the proportion of UHR expected to have reduced MMN may be relatively small and may depend strongly upon how the UHR sample are recruited. Further, even in those UHR who do transition the effect size for MMN reduction in first episode psychosis is lower than that seen in schizophrenia with a longer duration of illness [14].

None of the ERP measures provided support for our primary hypothesis that reduced MMN would be present during the prodromal period preceding onset of a first episode of psychosis. Trends in the data were either not significant or in the opposite direction to that predicted. Participants who transitioned to psychosis did not have smaller MMN 12 months before diagnosis than UHR who did not transition, nor were they smaller than those in a healthy comparison group. Our results differ from the conclusions of a recent meta-analysis [48] which found tentative support for reduced MMN in UHR who transitioned to psychosis compared to those who did not. However, our results are based upon an extremely small sample of only 6 UHT-T who provided EEG data at baseline. A power analysis, assuming a moderate effect size (0.5) and a transition rate of 10%, indicates that a sample of at least 35 UHR-T and 346 UHR-NT would be required to reliably detect a difference. Of previous reports, three found significantly reduced MMN in UHR-T compared to UHR-NT [33, 43, 44], one found a non-significant difference [45], and one found a difference for duration but not frequency MMN [42]. While the cumulative evidence to date appears to support the notion of reduced MMN in UHR-T, we agree with Bodatsch et al. [48] who argue more data are required before a definitive conclusion is drawn, especially with respect to the relative utility of different deviant types as predictors of transition in UHR groups.

We made two further predictions. First, that MMN to duration deviants would be more likely to predict transition to psychosis than MMN to frequency or intensity deviants. There were no interactions between deviant-type and group to support this prediction. This is the first study of UHR-T and UHR-NT to directly examine intensity MMN, and the third to examine frequency MMN [33, 42]. If we examine (non-significant) trends in our data, the unexpectedly larger MMN in UHR-T that UHR-NT was primarily due to differences in intensity, rather than duration MMN. To that extent, duration MMN was more consistent with the expectation of reduced MMN in UHR-T than was intensity MMN. Secondly, we predicted that MMN amplitude would show different developmental trajectories over time in those who did or did not transition to psychosis. Again, there were no significant effects in support of this assertion. However, our statistical power to detect any such change was even more severely limited by the small sample size (*n* = 5) of UHR-T who provided EEG data at both baseline and follow-up sessions.

Contrary to expectations, in our data, MMN tended to be of similar amplitude in UHR and controls, and larger in UHR-T than UHR-NT, with this latter difference reaching statistical significance when baseline and follow-up data were analysed together. These trends are inconsistent with the majority of MMN studies in UHR groups, but not with that from a study of MMN in 9–12 year old children considered to be at-risk [107]. This at-risk group consisted of children who presented with multiple putative antecedents of schizophrenia including speech or motor development problems; social, emotional, or behavioural problems; and psychotic-like experiences. Relative to matched controls, the at-risk group had larger duration MMN amplitudes. This similarly unexpected result was speculatively associated with abnormal neuroanatomical findings in an overlapping sample who had larger than expected grey matter and white matter volume in the left-temporal lobe [108], whereas volume reductions are more typically associated with schizophrenia [109]. We are currently undertaking a similar analysis of MRI structural data from the participants in this study.

MMN and P3a amplitude and latency were all affected by age. Our sample had an age range from 12 to 26 years. MMN and P3a amplitude *increased* and latency decreased with age most likely due to maturational changes during the teenage years. Other studies in healthy adolescents have found similar age effects for frequency MMN [110, 111], but in older age groups there are consistent reports of MMN amplitude *reductions* with age [29, 59] with maximal amplitudes seen in 20–30 year old adults. Within the UHR literature, age effects have been poorly reported, but some studies have reported adjusted MMN effects assuming a linear relationship between age and MMN amplitude [33, 41, 43]. For samples comprising adolescents and young adults, such as the present study, a linear model is adequate. However for samples spanning a larger age range, if the relationship between age and MMN is nonlinear, with amplitudes reaching a maximum in young adults, then linear corrections may not be the most appropriate. If MMN or P3a were to be adopted clinically as part of an at-risk assessment protocol, then standardisation of the measures for demographic variables, especially age, will be required [59].

There were no significant correlations between MMN and clinical measures within the UHR group at baseline that survived correction for multiple comparisons. As a trend, there was a reduction in MMN in UHR who meet the CAARMS criteria for attenuated psychotic symptoms relative to UHR who did not. Given that we observed no relationship between MMN and family history of mental illness, then this would be consistent with MMN reduction being a state marker of illness progression rather than a risk factor for schizophrenia per se.

In particular, MMN did not correlate with functional status or symptoms. The lack of relationship with positive or negative symptoms was not unexpected. Although in schizophrenia a weak correlation has been demonstrated within a particularly large sample [59], a meta-analysis by Umbricht and Krljes [13] reported that most studies found no correlation between MMN and either positive or negative symptoms. However, the relationship between MMN and functional status, especially GAF scores, is a well-replicated finding within schizophrenia [27, 112]. Other studies have reported no significant correlation between duration MMN and GAF in UHR [35, 39, 43, 104], but see [34]. The absence of this correlation within the UHR sample challenges the notion of UHR individuals being a homogenous group representative of those in an early stage of psychosis.

Unlike the MMN, there was a correlation between P3a amplitude and positive symptoms assessed by the CAARMS and with hallucinations assessed by the BPRS [37]. This dissociation between MMN and P3a appears consistent with the differential impact of current anti-psychotic medications on ERPs, which have minimal impact on MMN [113] but do modulate P3a amplitude [62, 63]. Anti-psychotics target dopaminergic pathways and primarily relieve positive symptoms [22]. While MMN may index NMDA receptor function within the glutamatergic neurotransmitter system [11, 22], the correlation between P3a and positive symptoms is consistent with reduced P3a being an index of dopaminergic dysfunction [62, 114].

Apart from the ERP measures, UHR and healthy controls differed on a broad range of demographic, clinical and neuropsychological measures. In particular, UHR had lower premorbid IQ, working memory, verbal memory, and executive functions. These results are consistent with a recent meta-analysis confirming cognitive deficits in UHR on multiple dimensions [115]. It suggests that the cognitive deficits linked to schizophrenia may be pre-existing risk factors for developing the disorder rather than symptoms of the disease progression. In contrast, there were relatively few differences between the transition and non-transition groups. Given the relatively small sample size in the UHR-T group and the large number of tests administered, these differences need to be interpreted with caution. UHR-T had poorer scores than UHR-NT for the CVLT-II verbal memory and CAARMS subjective emotional disturbance scales. Impairment of verbal memory as assessed by the CVLT, is recognised as a possible endophenotype for schizophrenia [116], but is also associated with cannabis use [116, 117]. Verbal memory has been demonstrated as one of the most impaired cognitive domains in UHR who transition to psychosis [115], in first-episode psychosis [118], and in schizophrenia [119], with impairments remaining stable over the course of the illness [120]. The other measure, subjective emotional disturbance, is one of six CAARMS sub-scales derived from Huber’s concept of *basic symptoms* [121]. Basic symptoms refer to a patient’s *self-awareness* of psychological impairments such as blunted or inappropriate affect, but differ from the concept of negative symptoms which refer to *observable signs* of impaired behaviours. Gross and Huber [122] argue that onset of basic symptoms precedes negative symptoms during the prodromal period whilst the patient retains sufficient cognitive resources to compensate for the self-perceived impairment. It has been reported that clusters of basic symptoms are highly predictive of transition to psychosis [122, 123]. However, reports on the predictive utility of subjective emotional disturbance are mixed with some studies showing some prognostic value [122, 124] and others none [123, 125]. Our data provide tentative support for greater inclusion of basic symptoms in the definition of UHR criteria as suggested by Nelson et al. [126] and supported by a recent meta-analysis of at-risk criteria [105].

Transition rates were lower than anticipated. Only seven of the 67 UHR available at the 12-month follow-up (10.4%) transitioned to psychosis. This is substantially lower than the original reports of ~40% transition rates reported in initial studies, but is consistent with a number of recent reports [105]. Wiltink et al. [49] have argued that there have been steadily declining transition rates in UHR studies which recruit using CAARMS criteria. In part, this reflects a selection bias. As more widespread mental health services become available to target youth at risk, individuals referred for UHR screening are being drawn from a broader community source with more heterogeneous symptomology, and individuals identified as UHR are being recruited earlier within the progression of their disorder. This seems consistent with the present study in which a moderately proactive recruitment strategy was adopted.

However, we need to acknowledge the relatively high drop-out rate in the study. Of the original 102 UHR participants, 34% withdrew or were lost to follow-up within the first 12 months with a quarter of these failing to complete baseline testing. We partly attribute this high drop-out rate to the extensive nature of the testing performed which required several days of commitment from participants. The LTFU group differed from other UHR on a number of measures at baseline. In particular they had higher rates of cannabis use, lower functional status and lower executive function, all of which are risk factors for transition to psychosis. Additionally, we noted a number of individuals who had especially poor functional status in their most recent assessment preceding being lost from the study. This suggests that some drop-outs were due to exacerbated clinical symptoms, possibly including a transition to psychosis. Other longitudinal studies have reported similar impairments in early psychosis groups who withdraw prior to follow-up testing [25, 127]. While it is not possible to determine the proportion of the LTFU group who may have transitioned to psychosis, it is likely that the overall transition rate was higher than that observed in the sub-sample who participated in both baseline and follow-up testing. However, baseline ERP amplitudes did not differ in the LTFU group compared to UHR who participated at follow-up, suggesting that drop-out rates may have had minimal effect on the ERP analyses.

The UHR group had a high rate of comorbidity for non-psychotic disorders, anxiety and depression being of primary concern. Irrespective of the relatively low transition rate to psychosis, this sub-group of help-seeking youth clearly appear to be in need of assistance from the mental health community. Notably, a quarter of the UHR sample had a history of attempted suicide or suicidal ideation. However, a significant portion of the sample were deemed to be in remission of UHR status one year after entry into the study, and even within those deemed to be still at risk, general measures of psychopathy, for example total BPRS scores, suggested substantive improvements in mental health as a general trend in most participants. In line with these observations, recent treatment guidelines for clients with an at risk mental state [128] emphasises treatment as usual for comorbid symptoms; recommends caution when applying this criteria to younger adolescents; and argues that pharmacological treatment *specifically* to prevent transition to psychosis is not justifiable based on current evidence.

In summary, we conducted a longitudinal prospective study into whether reduced MMN or P3a predicts transition to psychosis in youth at risk of developing psychosis as assessed with the CAARMS. Transition rates to psychosis were low within the limited time frame of the study. Consequently, the results need to be interpreted with caution and ideally combined with data from similar studies. Contrary to other recent studies and our predictions, MMN amplitudes tended to be larger in the transition than non-transition groups. There were relatively few correlations between MMN and clinically relevant measures, the exception being age, which should be treated as a confound when examining UHR and first episode psychosis samples. Although the majority of currently published studies support the finding of reduced MMN in UHR groups, our data suggests that the prognostic value of this deficit is still an open question and highlights some of the complexities associated with introducing this measure into clinical practice [129].

## Supporting information

S1 Table Demographic, clinical and neuropsychological measures at baseline. Means (Standard Deviation) and statistical significance of the difference between Controls and UHR after co-varying for age. For clarity, *p* values >.1 listed as n.s. Effect size reported as Cohen’s *d*.
(DOCX)

S2 Table Demographic, clinical and neuropsychological measures for UHR-T and UHR-NT at baseline. Means (Standard Deviation) and statistical comparison of UHR-T and UHR-NT. Statistics vary as applicable for data assessed (*F*: ANOVA with age as a covariate; *U*: Mann-Whitney *U* independent samples test; χ ^2^: Chi-square test; *t*: *t*-test). For clarity, *p* values >.1 listed as n.s. Effect size reported as Cohen’s *d*.
(DOCX)

S3 Table Correlations between MMN and Clinical Measures. Partial correlations, adjusting for age, between MMN peak amplitude at Fz and clinical measures within the UHR group at baseline. Note, since MMN is a negative potential, positive correlations indicate a reduction in MMN amplitude with increases in the covariate.
(DOCX)

S4 Table Correlations between P3a and Clinical Measures. Partial correlations, adjusting for age, between P3a peak amplitude at Fz and clinical measures within the UHR group at baseline.
(DOCX)

S5 Table Participants Lost To Follow-Up. Demographic, clinical and neuropsychological measures at baseline contrasting UHR who participated at the 12 month follow-up to those lost to follow-up. Means (Standard Deviation). Statistics vary as applicable for data assessed (*F*: ANOVA with age as a covariate; *U*: Mann-Whitney *U* independent samples test; χ ^2^: Chi-square test; *t*: *t*-test). For clarity, *p* values >.1 listed as n.s. Effect size reported as Cohen’s *d*.
(DOCX)
