# Supplementary material for: Electrophysiological, cognitive and clinical profiles of at-risk mental state: The longitudinal Minds in Transition (MinT) study
Source: PLoS One. 2017 Feb 10;12(2):e0171657. doi: 10.1371/journal.pone.0171657 (PMC5302824; doi:10.1371/journal.pone.0171657)
Supplement: S1 Table — Means (Standard Deviation) and statistical significance of the difference between Controls and UHR after co-varying for age. For clarity, p values >.1 listed as n.s. Effect size reported as Cohen’s d. (DOCX) [file pone.0171657.s001.docx]

***Supplementary Table 1.*** **Demographic, clinical and neuropsychological measures at baseline.** Means (Standard Deviation) and statistical significance of the difference between Controls and UHR after co-varying for age. For clarity, *p* values >.1 listed as n.s. Effect size reported as Cohen’s *d*.

| **Measure** | **Sub-Measure** | **Control** | **UHR** | **Statistic** | **Significance** | **Cohen’s *d*** |
| --- | --- | --- | --- | --- | --- | --- |
| *n* |  | 61 | 102 |  |  |  |
| Age |  | 19.1 (3.19) | 18.6 (2.71) | *t*(161)=1.04 | n.s. | 0.168 |
| Gender | Male | 32 | 47 | χ ^2^(1)=.62 | n.s. | 0.124 |
|  | Female | 29 | 55 |  |  |  |
| Handedness | Right | 48 | 83 | χ ^2^(2)=.30 | n.s. |  |
|  | Left | 5 | 7 |  |  |  |
|  | Ambidextrous | 6 | 8 |  |  |  |
| Accommodation | Family home | 37 | 61 | χ ^2^(1)=.01 | n.s. | 0.017 |
|  | Other | 24 | 41 |  |  |  |
| Employment | Employed/Student | 57 | 67 | χ ^2^(1)=21.9 | *p*<.001 ** | 0.799 |
|  | Unemployed | 1 | 34 |  |  |  |
| Years of Education |  | 11.9 (2.62) | 10.0 (2.54) | *F*(1,160)=26.0 | *p*<.001 ** | 0.807 |
| Medication |  |  |  |  |  |  |
|  | Nil | 52 | 50 | χ ^2^(1)=26.6 | *p*<.001 ** | 0.922 |
|  | Any Medication | 4 | 46 |  |  |  |
|  |  |  |  |  |  |  |
|  | Antidepressant |  | 32 |  |  |  |
|  | Stimulant |  | 6 |  |  |  |
|  | Benzodiazepine |  | 3 |  |  |  |
|  | Mood Stabiliser |  | 2 |  |  |  |
|  | Antipsychotic†† |  | 0 |  |  |  |
|  | Non psychotropic | 2 | 5 |  |  |  |
|  | Other | 2 | 8 |  |  |  |
| Previously Treated Mental Health Problems |  |  |  |  |  |  |
|  | Nil | 57 | 10 | χ ^2^(1)=114.2 | *p*<.001 ** | 3.193 |
|  | Any | 2 | 90 |  |  |  |
|  |  |  |  |  |  |  |
|  | Depression |  | 72 |  |  |  |
|  | Anxiety |  | 56 |  |  |  |
|  | Self-Harm |  | 37 |  |  |  |
|  | Drug/Alcohol |  | 35 |  |  |  |
|  | Suicide Attempt |  | 27 |  |  |  |
|  | Eating Disorder |  | 10 |  |  |  |
|  | Conduct/Violence |  | 10 |  |  |  |
|  | Obsessive Conduct |  | 10 |  |  |  |
|  | ADD |  | 8 |  |  |  |
|  | Pervasive Development | 1 | 7 |  |  |  |
|  | Mania |  | 6 |  |  |  |
|  | Early Psychosis |  | 4 |  |  |  |
|  | Other | 1 | 13 |  |  |  |
| Family History (First Degree) |  |  |  |  |  |  |
|  | Any Mental Health issue | 7 | 78 | χ ^2^(1)=65.2 | *p*<.001 ** | 1.667 |
|  | Nil | 52 | 22 |  |  |  |
|  |  |  |  |  |  |  |
|  | Schizophrenia |  | 24 | χ ^2^(1)=16.7 | *p*<.001 ** | 0.685 |
|  | Nil | 59 | 76 |  |  |  |
| Global Assessment of Functioning |  | 85.7 (6.12) | 55.5 (12.2) | *F*(1,145)=260 | *p*<.001 ** | 2.678 |
| Social and Occupational Function Assessment Scale |  | 85.2 (6.61) | 59.0 (13.1) | *F*(1,145)=171 | *p*<.001 ** | 2.176 |
| Global Functioning: Social |  | 8.83 (.854) | 6.29 (1.31) | *F*(1,153)=174 | *p*<.001 ** | 2.132 |
| Global Functioning: Role |  | 8.68 (.674) | 6.15 (1.31) | *F*(1,153)=187 | *p*<.001 ** | 2.211 |
| Drug Usage (Ever Used:Never Used) |  |  |  |  |  |  |
|  | Caffeine | 57:2 | 93:2 | χ ^2^(1)=.237 | n.s. | 0.079 |
|  | Alcohol | 50:11 | 87:9 | χ ^2^(1)=2.52 | n.s. | 0.255 |
|  | Tobacco | 21:40 | 74:21 | χ ^2^(1)=29.5 | *p*<.001 ** | 0.966 |
|  | Cannabis | 19:42 | 58:38 | χ ^2^(1)=12.8 | *p*<.001 ** | 0.596 |
|  | Hallucinogens | 7:53 | 38:58 | χ ^2^(1)=14.0 | *p*<.001 ** | 0.628 |
|  | Cocaine | 6:55 | 30:66 | χ ^2^(1)=9.68 | *p*=.002 ** | 0.513 |
|  | Amphetamines | 6:55 | 29:67 | χ ^2^(1)=8.94 | *p*=.003 ** | 0.492 |
|  | Inhalants/Solvents | 2:59 | 18:78 | χ ^2^(1)=8.03 | *p*=.005 ** | 0.464 |
|  | Tranquilisers | 2:58 | 18:78 | χ ^2^(1)=7.85 | *p*=.005 ** | 0.460 |
|  | Other Opiates | 0:61 | 11:85 | χ ^2^(1)=7.52 | *p*=.006 ** | 0.449 |
|  | Heroin | 0:61 | 7:89 | χ ^2^(1)=4.67 | *p*=.031 * | 0.350 |
|  | Barbiturates | 0:61 | 0:96 | n.a. |  |  |
| AUDIT |  | 5.27 (5.08) | 8.28 (8.00) | *F*(1,154)=7.47 | *p*=.007 ** | 0.440 |
| CUDIT |  | 1.55 (5.60) | 7.66 (14.8) | *F*(1,153)=10.7 | *p*=.001 ** | 0.530 |
| Cannabis Use |  |  |  |  |  |  |
|  | Age First Used | 16.5 (1.81) | 14.8 (2.27) | *F*(1,73)=7.60 | *p*=.007 ** | 0.645 |
|  | Age Regular Usage | 16.6 (3.21) | 15.2 (1.72) | *F*(1,39)=1.78 | *p*=.193 | 0.425 |
|  | Duration Use (users) | 2.58 (2.59) | 3.50 (2.62) | *F*(1,74)=6.61 | *p*=.012 * | 0.598 |
|  | Duration Use (all) | .804 (1.86) | 2.12 (2.66) | *F*(1,154)=17.0 | *p*<.001 ** | 0.664 |
| †Schizotypal Personality Questionnaire |  | 8.97 (7.86) | 34.26 (16.6) | *F*(1,130)=75.2 | *p*<.001 ** | 1.521 |
| †Rosenberg Self Esteem Scale |  | 20.4 (4.71) | 15.5 (6.69) | *F*(1,132)=17.0 | *p*<.001 ** | 0.718 |
| †Beck Depression Inventory II |  | 4.62 (4.64) | 22.1 (13.0) | *F*(1,132)=61.7 | *p*<.001 ** | 1.368 |
| †Beck Anxiety Inventory |  | 4.16 (3.64) | 18.4 (11.9) | *F*(1,134)=51.2 | *p*<.001 ** | 1.236 |
| †Eysenck Personality Questionnaire – Revised |  | 2.37 (1.68) | 7.66 (3.29) | *F*(1,132)=87.0 | *p*<.001 ** | 1.623 |
| †Pittsburgh Sleep Quality Index | Global Score | 4.42 (2.64) | 7.77 (3.90) | *F*(1,110)=16.4 | *p*<.001 ** | 0.771 |
| †University of Pennsylvania Smell Identification Task |  | 34.1 (3.53) | 32.9 (3.44) | *F*(1,128)=2.15 | n.s. | 0.259 |
| WASI 2 Subscale IQ |  | 118 (12.0) | 104 (16.5) | *F*(1,153)=34.2 | *p*<.001 ** | 0.946 |
| †Weschler Memory Scale III (Scaled) |  |  |  |  |  |  |
|  | Letter Number Sequencing | 11.0 (2.60) | 9.79 (2.75) | *F*(1,124)=4.88 | *p*=.029 * | 0.397 |
|  | Digit Span Total | 11.4 (3.19) | 9.79 (2.69) | *F*(1,122)=6.96 | *p*=.009 ** | 0.478 |
| California Verbal Learning Task II (Scaled) |  |  |  |  |  |  |
|  | Immediate Recall | 55.9 (8.47) | 50.8 (12.8) | *F*(1,148)=7.07 | *p*=.009 ** | 0.437 |
|  | Mean Delayed Recall | .223 (.855) | -.167 (1.05) | *F*(1,147)=5.47 | *p*=.021 * | 0.386 |
|  | Recognition | -.138 (.857) | -.330 (.827) | *F*(1,146)=1.91 | n.s. | 0.229 |
| DKEFS Trail Making (Scaled) | C4 Number Letter Sequencing | 10.3 (2.34) | 9.01 (1.98) | *F*(1,153)=7.50 | *p*=.007 ** | 0.443 |
| DKEFS Verbal Fluency (Scaled) | C3 Category Switching Accuracy | 13.7 (2.39) | 12.2 (2.76) | *F*(1,153)=12.2 | *p*=.001 ** | 0.564 |
| DKEFS Tower Test (Scaled) | Total Achievement | 10.5 (2.45) | 9.84 (2.29) | *F*(1,153)=2.67 | n.s. | 0.264 |
| †DKEFS Colour Word Interference (Scaled) | C3 Inhibition | 11.7 (2.16) | 9.29 (3.25) | *F*(1,132)=16.1 | *p*<.001 ** | 0.698 |
| Visual Patterns Test |  | 10.6 (1.78) | 8.41 (2.21) | *F*(1,140)=38.2 | *p*<.001 ** | 1.045 |
| Hinting Task |  | 18.4 (1.77) | 17.1 (2.56) | *F*(1,150)=12.2 | *p*=.001 ** | 0.570 |
| Picture Sequencing Task | TOM Total | 21.2 (3.56) | 20.1 (4.07) | *F*(1,151)=2.77 | p=.098 | 0.271 |
| Reading the Mind in the Eyes |  | 21.2 (2.57) | 21.1 (2.89) | *F*(1,150)=.008 | n.s. | 0.014 |

†Only a sub-set of 38 control participants completed the indicated clinical tests. This subgroup was older than the UHR group (19.6 vs 18.7 yrs, *t*(132)=1.80, *p*=.074).
†† Antipsychotic medication was an exclusion criterion.
* *p*<.05 uncorrected
** *p*<.01 uncorrected
