# Supplementary material for: Electrophysiological, cognitive and clinical profiles of at-risk mental state: The longitudinal Minds in Transition (MinT) study
Source: PLoS One. 2017 Feb 10;12(2):e0171657. doi: 10.1371/journal.pone.0171657 (PMC5302824; doi:10.1371/journal.pone.0171657)
Supplement: S2 Table — Means (Standard Deviation) and statistical comparison of UHR-T and UHR-NT. Statistics vary as applicable for data assessed (F: ANOVA with age as a covariate; U: Mann-Whitney U independent samples test; χ 2: Chi-square test; t: t-test). For clarity, p values >.1 listed as n.s. Effect size reported as Cohen’s d. (DOCX) [file pone.0171657.s002.docx]

***Supplementary Table 2.*** **Demographic, clinical and neuropsychological measures for UHR-T and UHR-NT at baseline.** Means (Standard Deviation) and statistical comparison of UHR-T and UHR-NT. Statistics vary as applicable for data assessed (*F*: ANOVA with age as a covariate; *U:* Mann-Whitney *U* independent samples test; χ ^2^: Chi-square test; *t*: *t*-test). For clarity, *p* values >.1 listed as n.s. Effect size reported as Cohen’s *d*.

| **Measure** | **Sub-Measure** | **UHR-NT** | **UHR-T** | **Statistic** | **Significance** | **Cohen’s d** |
| --- | --- | --- | --- | --- | --- | --- |
| *n* |  | 60 | 7 |  |  |  |
| Age |  | 18.4 (2.62) | 19.8 (1.96) | t(65)=1.35 | n.s. | 0.539 |
| Gender | Male | 27 | 4 | χ ^2^(1)=.372 | n.s. | 0.149 |
|  | Female | 33 | 3 |  |  |  |
| Handedness | Right | 53 | 5 | χ ^2^(2)=2.34 | n.s. |  |
|  | Left | 4 | 1 |  |  |  |
|  | Ambidextrous | 2 | 1 |  |  |  |
| Accommodation | Family home | 40 | 4 | χ ^2^(1)=.252 | n.s. | 0.123 |
|  | Other | 20 | 3 |  |  |  |
| Employment | Employed/Student | 42 | 3 | χ ^2^(1)=2.31 | n.s. | 0.381 |
|  | Unemployed | 17 | 4 |  |  |  |
| Education (Years) |  | 10.1 (2.69) | 10.4 (2.56) | *F*(1,64)=.59 | n.s. | 0.191 |
| Current Medication |  |  |  |  |  |  |
|  | Any Medication | 28 | 4 | χ ^2^(1)=.127 | n.s. | 0.090 |
|  | Nil | 28 | 3 |  |  |  |
| Previously Treated Mental Health Problems |  |  |  |  |  |  |
|  | Any Previous | 52 | 5 | χ ^2^(1)=1.48 | n.s. | 0.303 |
|  | Nil | 7 | 2 |  |  |  |
| Family History (1^st^ degree) |  |  |  |  |  |  |
|  | Any Mental Health Issue | 44 | 4 | χ ^2^(1)=.96 | n.s. | 0.243 |
|  | Nil | 15 | 3 |  |  |  |
|  |  |  |  |  |  |  |
|  | Schizophrenia | 11 | 2 | χ ^2^(1)=.39 | n.s. | 0.154 |
|  | No Schizophrenia | 48 | 5 |  |  |  |
| Global Assessment of Functioning |  | 57.4 (12.2) | 52.7 (13.4) | *F*(1,64)=.88 | n.s. | 0.234 |
| Social and Occupational Function Assessment Scale |  | 61.8 (12.4) | 53.4 (11.6) | *F*(1,64)=3.22 | *p*=.077 | 0.449 |
| Global Functioning: Social |  | 6.54 (1.26) | 6.21 (1.19) | *F*(1,62)=.38 | n.s. | 0.158 |
| Global Functioning: Role |  | 6.54 (.961) | 5.57 (1.51) | *F*(1,62)=6.04 | *p*=.017 * | 0.624 |
| CAARMS (Frequency Weighted) |  |  |  |  |  |  |
|  | Positive Symptoms | 9.03 (4.84) | 10.9 (3.85) | *F*(1,64)=.97 | n.s. | 0.247 |
|  | Negative Symptoms | 7.81 (6.38) | 9.81 (8.62) | *F*(1,64)=.37 | *n.s.* | 0.151 |
|  | Cognitive Change | 9.65 (7.52) | 15.0 (5.90) | *F*(1,63)=2.96 | *p*=.090 | 0.448 |
|  | Emotional Disturbance | 7.53 (6.85) | 17.8 (7.22) | *F*(1,63)=11.4 | *p*=.001 ** | 0.849 |
|  | Behavioural Change | 6.94 (5.58) | 8.32 (5.92) | *F*(1,63)=.16 | n.s. | 0.100 |
|  | Motor/Physical Change | 3.32 (3.78) | 2.56 (2.30) | *F*(1,63)=.20 | n.s. | 0.113 |
|  | General Psychopathology | 6.37 (4.50) | 9.13 (5.04) | *F*(1,63)=2.34 | n.s. | 0.385 |
| BPRS | Total | 44.3 (10.3) | 52.0 (12.4) | *F*(1,61)=3.14 | *p*=.082 | 0.453 |
| Drug Usage (Ever Used:Never Used) |  |  |  |  |  |  |
|  | Caffeine | 56:1 | 7:0 | χ ^2^(1)=.125 | n.s. | 0.089 |
|  | Alcohol | 49:8 | 7:0 | χ ^2^(1)=1.12 | n.s. | 0.267 |
|  | Tobacco | 38:18 | 6:1 | χ ^2^(1)=.942 | n.s | 0.246 |
|  | Cannabis | 27:30 | 5:2 | χ ^2^(1)=1.44 | n.s. | 0.303 |
|  | Hallucinogens | 19:38 | 2:5 | χ ^2^(1)=.064 | n.s. | 0.063 |
|  | Cocaine | 16:41 | 2:5 | χ ^2^(1)=.001 | n.s. | 0.008 |
|  | Amphetamines | 13:44 | 2:5 | χ ^2^(1)=.115 | n.s. | 0.085 |
|  | Inhalants | 10:47 | 2:5 | χ ^2^(1)=.498 | n.s. | 0.177 |
|  | Tranquilisers | 7:50 | 2:5 | χ ^2^(1)=1.37 | n.s. | 0.296 |
|  | Other Opiates | 5:52 | 0:7 | χ ^2^(1)=.666 | n.s. | 0.205 |
|  | Heroin | 1:56 | 1:6 | χ ^2^(1)=3.23 | *p*=.072 | 0.461 |
|  | Barbiturates | 0:57 | 0:7 | n/a |  |  |
| AUDIT |  | 7.36 (7.87) | 9.86 (8.44) | *U*(7,59)=165.5, *r*=.106 | n.s. | 0.213 |
| CUDIT |  | 4.85 (10.2) | 8.57 (14.7) | *U*(7,60)=201.5, *r*=.026 | n.s. | 0.026 |
| Cannabis Use |  |  |  |  |  |  |
|  | Age First Used | 14.8 (2.42) | 15.3 (2.22) | *F*(1,28)=.060 | n.s. | 0.089 |
|  | Age Regular Use | 15.2 (1.63) | 15.0 (2.83) | *F*(1,19)=.040 | n.s. | 0.091 |
|  | Duration Use (users) | 3.22 (2.63) | 2.77 (2.17) | *F*(1,29)=.590 | n.s. | 0.285 |
|  | Duration Use (all) | 1.52 (2.41) | 1.98 (2.23) | *F*(1,61)=.001 | n.s. | 0.006 |
| Schizotypal Personality Questionnaire |  | 32.8 (15.8) | 30.4 (19.7) | *F*(1,63)=.013 | n.s. | 0.029 |
| Rosenberg Self Esteem Scale |  | 15.3 (6.87) | 16.7 (6.97) | *F*(1,63)=.142 | n.s. | 0.095 |
| Beck Depression Inventory II |  | 20.7 (13.1) | 26.7 (14.0) | *F*(1,64)=1.72 | n.s. | 0.327 |
| Beck Anxiety Inventory |  | 16.6 (10.2) | 19.3 (13.8) | *F*(1,64)=.441 | n.s. | 0.166 |
| Eysenck Personality Questionnaire – Revised |  | 7.37 (3.29) | 7.57 (3.82) | *F*(1,63)=.080 | n.s. | 0.071 |
| Pittsburgh Sleep Quality Index | Global Score | 7.88 (3.74) | 6.60 (3.21) | *F*(1,52)=.317 | n.s. | 0.156 |
| University of Pennsylvania Smell Identification Task |  | 32.6 (3.48) | 35.5 (2.59) | *F*(1,61)=3.24 | *p*=.077 | 0.461 |
| WASI 2 Subscale IQ |  | 105 (16.6) | 104 (19.8) | *F*(1,63)=.204 | n.s. | 0.114 |
| Weschler Memory Scale III (Scaled) |  |  |  |  |  |  |
|  | Letter Number Sequencing | 9.79 (2.72) | 11.6 (3.31) | *F*(1,61)=2.17 | n.s. | 0.377 |
|  | Digit Span Total | 9.84 (2.50) | 11.7 (3.55) | *F*(1,61)=2.30 | n.s. | 0.389 |
| California Verbal Learning Task II (Std. Scores) |  |  |  |  |  |  |
|  | Immediate Recall | 53.0 (11.9) | 47.3 (14.1) | *F*(1,61)=3.10 | *p*=.083 | 0.451 |
|  | Mean Delayed Recall | -.022 (.880) | -.857 (1.79) | *F*(1,61)=4.99 | *p*=.029 * | 0.572 |
|  | Recognition | -.242 (.696) | -1.29 (1.82) | *F*(1,62)=8.29 | *p*=.005 ** | 0.731 |
| DKEFS Trail Making (Scaled) | C4 Number Letter Sequencing | 9.36 (3.11) | 10.0 (2.58) | *F*(1,63)=.131 | n.s. | 0.091 |
| DKEFS Verbal Fluency (Scaled) | C3 Category Switching Accuracy | 12.9 (2.68) | 11.7 (2.22) | *F*(1,64)=.953 | n.s. | 0.244 |
| DKEFS Tower Test (Scaled) | Total Achievement | 10.1 (2.22) | 9.71 (2.22) | *F*(1,63)=.219 | n.s. | 0.118 |
| DKEFS Colour Word Interference (Scaled) | C3 Inhibition | 9.20 (3.54) | 9.14 (2.41) | *F*(1,64)=.011 | n.s. | 0.027 |
| Visual Patterns Test |  | 8.43 (2.31) | 8.99 (2.24) | *F*(1,58)=.123 | n.s. | 0.092 |
| Hinting Task |  | 17.1 (2.69) | 17.0 (2.97) | *F*(1,62)=.228 | n.s. | 0.121 |
| Picture Sequencing Task | TOM Total | 20.5 (3.80) | 20.3 (5.74) | *F*(1,63)=.011 | n.s. | 0.026 |
| Reading the Mind in the Eyes |  | 21.2 (2.94) | 19.7 (2.14) | *F*(1,63)=2.23 | n.s. | 0.376 |

* *p*<.05 uncorrected
** *p*<.01 uncorrected
