# Supplementary material for: Electrophysiological, cognitive and clinical profiles of at-risk mental state: The longitudinal Minds in Transition (MinT) study
Source: PLoS One. 2017 Feb 10;12(2):e0171657. doi: 10.1371/journal.pone.0171657 (PMC5302824; doi:10.1371/journal.pone.0171657)
Supplement: S3 Table — Partial correlations, adjusting for age, between MMN peak amplitude at Fz and clinical measures within the UHR group at baseline. Note, since MMN is a negative potential, positive correlations indicate a reduction in MMN amplitude with increases in the covariate. (DOCX) [file pone.0171657.s003.docx]

***Supplementary Table 3.*** **Partial correlations, adjusting for age, between MMN peak amplitude at Fz and clinical measures within the UHR group at baseline.** Note, since MMN is a negative potential, positive correlations indicate a reduction in MMN amplitude with increases in the covariate. MMN_Mean_ = (MMN_Dur_ + MMN_Frq_ + MMN_Int_)/3.

|  | *n* | MMN_Dur_ | MMN_Frq_ | MMN_Int_ | MMN_Mean_ |
| --- | --- | --- | --- | --- | --- |
| †Age | 80 | -.127 | -.020 | -.253 * | -.170 |
|  |  |  |  |  |  |
| GAF | 76 | -.092 | .051 | .064 | -.001 |
| SOFAS | 76 | -.097 | .031 | .060 | -.012 |
| Global Functioning – Social | 74 | -.121 | -.027 | .034 | -.053 |
| Global Functioning - Role | 74 | -.049 | -.004 | .116 | .022 |
|  |  |  |  |  |  |
| CAARMS |  |  |  |  |  |
| Total | 72 | -.070 | -.165 | -.137 | -.143 |
| Positive Symptoms | 77 | .033 | -.193 | -.069 | -.076 |
| Cognitive Change | 75 | .026 | -.127 | -.165 | -.097 |
| Emotional Disturbance | 74 | -.015 | -.114 | -.083 | -.079 |
| Negative Symptoms | 75 | -.135 | -.069 | -.069 | -.115 |
| Behavioural Change | 74 | -.119 | -.081 | -.081 | -.116 |
| Motor/Physical Change | 75 | -.006 | -.061 | -.068 | -.045 |
| General Psychopathology | 74 | -.093 | -.227 * | -.142 | -.177 |
|  |  |  |  |  |  |
| BPRS (Total) | 70 | .033 | -.106 | -.063 | -.045 |
|  |  |  |  |  |  |
| SPQ (Total) | 76 | .012 | -.109 | -.049 | -.050 |
| RSES | 76 | .070 | .135 | -.120 | -.030 |
| BDI-II | 76 | -.160 | -.273 * | -.183 | -.240 * |
| BAI | 77 | .007 | -.035 | -.009 | -.012 |
| EPQ-R | 76 | -.062 | -.170 | -.050 | -.106 |
|  |  |  |  |  |  |
| CUDIT | 76 | .194 | .211 | .215 | .249 * |
| Age first used Cannabis | 41 | .017 | .053 | .145 | .086 |
| Time since last use Cannabis | 75 | .180 | .101 | .059 | .144 |

†Pearson Correlation
* *p*<.05 uncorrected
** *p*<.01 uncorrected
