# Supplementary material for: Electrophysiological, cognitive and clinical profiles of at-risk mental state: The longitudinal Minds in Transition (MinT) study
Source: PLoS One. 2017 Feb 10;12(2):e0171657. doi: 10.1371/journal.pone.0171657 (PMC5302824; doi:10.1371/journal.pone.0171657)
Supplement: S4 Table — Partial correlations, adjusting for age, between P3a peak amplitude at Fz and clinical measures within the UHR group at baseline. (DOCX) [file pone.0171657.s004.docx]

***Supplementary Table 4.*** **Partial correlations, adjusting for age, between P3a peak amplitude at Fz and clinical measures within the UHR group at baseline.** P3a_Mean_ = (P3a_Dur_ + P3a_Frq_ + P3a_Int_)/3.

|  | *n* | P3a_Dur_ | P3a_Frq_ | P3a_Int_ | P3a_Mean_ |
| --- | --- | --- | --- | --- | --- |
| †Age | 80 | .221 * | .194 | -.065 | .154 |
|  |  |  |  |  |  |
| GAF | 76 | -.060 | .018 | .082 | .014 |
| SOFAS | 76 | .067 | .078 | .099 | .100 |
| Global Functioning – Social | 74 | .076 | .134 | .111 | .132 |
| Global Functioning - Role | 74 | .050 | .034 | .013 | .041 |
|  |  |  |  |  |  |
| CAARMS |  |  |  |  |  |
| Total | 72 | .014 | .028 | -.081 | -.013 |
| Positive Symptoms | 77 | -.189 | -.173 | -.313 ** | -.275 * |
| Cognitive Change | 75 | -.001 | -.011 | -.030 | -.017 |
| Emotional Disturbance | 74 | -.014 | -.030 | .016 | -.013 |
| Negative Symptoms | 75 | .170 | .096 | .016 | .120 |
| Behavioural Change | 74 | -.017 | .109 | .021 | .048 |
| Motor/Physical Change | 75 | .025 | .029 | -.120 | -.023 |
| General Psychopathology | 74 | .026 | .085 | -.053 | .028 |
|  |  |  |  |  |  |
| BPRS (Total) | 70 | -.017 | -.004 | -.120 | -.091 |
|  |  |  |  |  |  |
| SPQ (Total) | 76 | -.079 | -.033 | -.237 * | -.138 |
| RSES | 76 | -.047 | .102 | .061 | .049 |
| BDI-II | 76 | -.038 | -.012 | -.045 | -.038 |
| BAI | 77 | -.218 | -.097 | -.147 | -.190 |
| EPQ-R | 76 | -.106 | -.054 | -.147 | -.125 |
|  |  |  |  |  |  |
| CUDIT | 76 | -.128 | -.093 | -.026 | -.104 |
| Age first used Cannabis | 41 | .114 | -.170 | -.053 | -.047 |
| Time since last use Cannabis | 75 | -.153 | .107 | .005 | -.013 |

†Pearson Correlation
* *p*<.05 uncorrected
** *p*=.005 uncorrected
