# Supplementary material for: Electrophysiological, cognitive and clinical profiles of at-risk mental state: The longitudinal Minds in Transition (MinT) study
Source: PLoS One. 2017 Feb 10;12(2):e0171657. doi: 10.1371/journal.pone.0171657 (PMC5302824; doi:10.1371/journal.pone.0171657)
Supplement: S5 Table — Demographic, clinical and neuropsychological measures at baseline contrasting UHR who participated at the 12 month follow-up to those lost to follow-up. Means (Standard Deviation). Statistics vary as applicable for data assessed (F: ANOVA with age as a covariate; U: Mann-Whitney U independent samples test; χ 2: Chi-square test; t: t-test). For clarity, p values >.1 listed as n.s. Effect size reported as Cohen’s d. (DOCX) [file pone.0171657.s005.docx]

***Supplementary Table 5.*** **Demographic, clinical and neuropsychological measures at baseline contrasting UHR who participated at the 12 month follow-up to those lost to follow-up.** Means (Standard Deviation). Statistics vary as applicable for data assessed (*F*: ANOVA with age as a covariate; *U:* Mann-Whitney *U* independent samples test; χ ^2^: Chi-square test; *t*: *t*-test). For clarity, *p* values >.1 listed as n.s. Effect size reported as Cohen’s *d*.

| **Measure** | **Sub-Measure** | **Participated** | **LTFU** | **Statistic** | **Significance** | **Cohen’s d** |
| --- | --- | --- | --- | --- | --- | --- |
| *n* |  | 67 | 35 |  |  |  |
| Age |  | 18.5 (2.58) | 18.8 (2.97) | *t*(100)=.44 | n.s. | 0.110 |
| Gender | Male | 31 | 16 | χ ^2^(1)=.003 | n.s. | 0.011 |
|  | Female | 36 | 19 |  |  |  |
| Handedness | Right | 58 | 25 | χ ^2^(2)=3.54 | n.s. |  |
|  | Left | 5 | 2 |  |  |  |
|  | Ambidextrous | 3 | 5 |  |  |  |
| Accommodation | Family home | 45 | 20 | χ ^2^(1)=1.00 | n.s. | 0.199 |
|  | Other | 22 | 15 |  |  |  |
| Employment | Employed/Student | 45 | 22 | χ ^2^(1)=.290 | n.s. | 0.107 |
|  | Unemployed | 21 | 13 |  |  |  |
| Education (Years) |  | 10.1 (2.66) | 9.96 (2.34) | *F*(1,99)=.426 | n.s. | 0.131 |
| Current Medication |  |  |  |  |  |  |
|  | Nil | 31 | 19 | χ ^2^(1)=.608 | n.s. | 0.160 |
|  | Any Medication | 32 | 14 |  |  |  |
| Previous Mental Health Problems |  |  |  |  |  |  |
|  | Nil | 9 | 1 | χ ^2^(1)=2.85 | *p*=.091 | 0.343 |
|  | Any Previous | 57 | 33 |  |  |  |
| Family History |  |  |  |  |  |  |
|  | 1^st^ any Mental Health Issue | 48 | 30 | χ ^2^(1)=3.15 | *p*=.076 | 0.360 |
|  | 1^st^ Nil | 18 | 4 |  |  |  |
|  |  |  |  |  |  |  |
|  | 1^st^ Schizophrenia | 13 | 11 | χ ^2^(1)=1.97 | n.s. | 0.284 |
|  | 1^st^ No Schizophrenia | 53 | 23 |  |  |  |
| Global Assessment of Functioning |  | 56.9 (12.3) | 52.7 (11.6) | *F*(1,96)=2.61 | n.s. | 0.330 |
| Social and Occupational Function Assessment Scale |  | 60.9 (12.5) | 54.9 (13.6) | *F*(1,96)=4.80 | *p*=.031 * | 0.447 |
| Global Functioning: Social |  | 6.51 (1.25) | 5.84 (1.32) | *F*(1,94)=5.87 | *p*=.017 * | 0.500 |
| Global Functioning: Role |  | 6.44 (1.06) | 5.56 (1.55) | *F*(1,94)=11.1 | *p*=.001 ** | 0.687 |
| CAARMS (Frequency Weighted) |  |  |  |  |  |  |
|  | Positive Symptoms | 9.22 (4.75) | 7.67 (5.08) | *F*(1,99)=2.67 | n.s. | 0.329 |
|  | Negative Symptoms | 8.01 (6.60) | 8.55 (7.97) | *F*(1,96)=.142 | n.s. | 0.077 |
|  | Cognitive Change | 10.1 (7.51) | 9.94 (6.56) | *F*(1,96)=.020 | n.s. | 0.029 |
|  | Emotional Disturbance | 8.47 (7.45) | 8.41 (7.97) | *F*(1,95)=.002 | n.s. | 0.010 |
|  | Behavioural Change | 7.09 (5.58) | 7.39 (7.22) | *F*(1,94)=.058 | n.s. | 0.050 |
|  | Motor/Physical Change | 3.25 (3.66) | 3.17 (4.38) | *F*(1,96)=.017 | n.s. | 0.027 |
|  | General Psychopathology | 6.67 (4.60) | 6.62 (4.81) | *F*(1,92)=.003 | n.s. | 0.011 |
| BPRS | Total | 45.2 (10.7) | 41.6 (9.02) | *F*(1,89)=2.37 | n.s. | 0.327 |
| Drug Usage (Ever Used:Never Used) |  |  |  |  |  |  |
|  | Caffeine | 63:1 | 30:1 | χ ^2^(1)=.280 | n.s. | 0.109 |
|  | Alcohol | 56:8 | 31:1 | χ ^2^(1)=2.21 | n.s. | 0.307 |
|  | Tobacco | 44:19 | 30:2 | χ ^2^(1)=7.05 | *p*=.008 ** | 0.566 |
|  | Cannabis | 32:32 | 26:6 | χ ^2^(1)=8.71 | *p*=.003 ** | 0.632 |
|  | Hallucinogens | 21:43 | 17:15 | χ ^2^(1)=3.68 | *p*=.055 | 0.399 |
|  | Cocaine | 18:46 | 12:20 | χ ^2^(1)=.873 | n.s. | 0.192 |
|  | Amphetamines | 15:49 | 14:18 | χ ^2^(1)=4.18 | *p*=.041 * | 0.427 |
|  | Inhalants | 12:52 | 6:26 | χ ^2^(1)=.000 | n.s. | 0.000 |
|  | Tranquilisers | 9:55 | 9:23 | χ ^2^(1)=2.77 | *p*=.096 | 0.345 |
|  | Other Opiates | 5:59 | 6:26 | χ ^2^(1)=2.52 | n.s. | 0.328 |
|  | Heroin | 2:62 | 5:27 | χ ^2^(1)=4.93 | *p*=.026 * | 0.465 |
|  | Barbiturates | 0:64 | 0:32 | n.a. |  |  |
| AUDIT |  | 7.62 (7.90) | 9.62 (8.15) | *U*(32,66)=917, *Z*=1.06 | n.s. | 0.215 |
| CUDIT |  | 5.24 (10.7) | 12.9 (20.3) | *U*(31,67)=818, *Z*=1.95 | *p*=.051 | 0.402 |
| Cannabis Use |  |  |  |  |  |  |
|  | Age First Used | 14.9 (2.36) | 14.7 (2.20) | *F*(1,54)=.138 | n.s. | 0.101 |
|  | Age Regular Use | 15.1 (1.67) | 15.3 (1.84) | *F*(1,34)=.076 | n.s. | 0.094 |
| Schizotypal Personality Questionnaire |  | 32.5 (16.1) | 38.1 (17.4) | *F*(1,93)=2.44 | n.s. | 0.324 |
| Rosenberg Self Esteem Scale |  | 15.4 (6.84) | 15.5 (6.47) | *F*(1,94)=..004 | n.s. | 0.013 |
| Beck Depression Inventory II |  | 21.4 (13.2) | 23.8 (12.5) | *F*(1,95)=.705 | n.s. | 0.172 |
| Beck Anxiety Inventory |  | 16.9 (10.5) | 21.6 (14.0) | *F*(1,96)=3.38 | *p*=.069 | 0.375 |
| Eysenck Personality Questionnaire – Revised |  | 7.39 (3.32) | 8.23 (3.22) | *F*(1,94)=1.28 | n.s. | 0.234 |
| Pittsburgh Sleep Quality Index | Global Score | 7.76 (3.69) | 7.78 (4.38) | *F*(1,79)=.005 | n.s. | 0.016 |
| University of Pennsylvania Smell Identification Task |  | 32.9 (3.50) | 32.8 (3.38) | *F*(1,90)=.054 | n.s. | 0.049 |
| WASI 2 Subscale IQ |  | 105 (16.8) | 100 (15.8) | *F*(1,93)=1.90 | n.s. | 0.286 |
| Weschler Memory Scale III (Scaled) |  |  |  |  |  |  |
|  | Letter Number Sequencing | 9.98 (2.81) | 9.31 (2.59) | *F*(1,87)=1.49 | n.s. | 0.262 |
|  | Digit Span Total | 10.1 (2.66) | 9.12 (2.68) | *F*(1,86)=3.47 | *p*=.066 | 0.402 |
| California Verbal Learning Task II (Std. Scores) |  |  |  |  |  |  |
|  | Immediate Recall | 52.4 (12.2) | 47.1 (13.6) | *F*(1,88)=4.02 | *p*=.048 * | 0.427 |
|  | Mean Delayed Recall | -.113 (1.03) | -.296 (1.10) | *F*(1,88)=.665 | n.s. | 0.174 |
|  | Recognition | -.355 (.921) | -.269 (.533) | *F*(1,88)=.241 | n.s. | 0.105 |
| DKEFS Trail Making (Scaled) | C4 Number Letter Sequencing | 9.42 (3.05) | 8.10 (2.66) | *F*(1,93)=4.15 | *p*=.045 * | 0.422 |
| DKEFS Verbal Fluency (Scaled) | C3 Category Switching Accuracy | 12.8 (2.64) | 11.0 (2.65) | *F*(1,93)=8.71 | *p*=.004 ** | 0.612 |
| DKEFS Tower Test (Scaled) | Total Achievement | 10.1 (2.21) | 9.40 (2.44) | *F*(1,93)=1.40 | n.s. | 0.246 |
| DKEFS Colour Word Interference (Scaled) | C3 Inhibition | 9.19 (3.43) | 9.50 (2.85) | *F*(1,94)=.136 | n.s. | 0.076 |
| Visual Patterns Test |  | 8.50 (2.29) | 8.20 (2.04) | *F*(1,82)=.543 | n.s. | 0.163 |
| Hinting Task |  | 17.1 (2.70) | 17.0 (2.24) | *F*(1,89)=.344 | n.s. | 0.124 |
| Picture Sequencing Task | TOM Total | 20.5 (3.99) | 19.3 (4.22) | *F*(1,90)=1.36 | n.s. | 0.246 |
| Reading the Mind in the Eyes |  | 21.0 (2.88) | 21.5 (2.94) | *F*(1,89)=.272 | n.s. | 0.111 |

* *p*<.05 uncorrected
** *p*<.01 uncorrected
